# Individualized lipid profile in urine-derived extracellular vesicles from clinical patients with *Mycobacterium tuberculosis* infections

**DOI:** 10.3389/fmicb.2024.1409552

**Published:** 2024-05-30

**Authors:** Lingna Lyu, Hongyan Jia, Qiuyue Liu, Wenxia Ma, Zihui Li, Liping Pan, Xiuli Zhang

**Affiliations:** ^1^Department of Gastroenterology and Hepatology, Beijing You’an Hospital Affiliated to Capital Medical University, Beijing, China; ^2^Beijing Key Laboratory for Drug Resistant Tuberculosis Research, Beijing Chest Hospital Affiliated to Capital Medical University, Beijing Tuberculosis and Thoracic Tumor Research Institute, Beijing, China; ^3^The Chinese Academy of Sciences (CAS) Center for Excellence in Nanoscience, National Center for Nanoscience and Technology of China, Beijing, China

**Keywords:** *Mycobacterium tuberculosis*, extracellular vesicles, lipidome, proteome, biomarker, lipid raft, immune response

## Abstract

**Background:**

Lipids are a key nutrient source for the growth and reproduction of *Mycobacterium tuberculosis* (*Mtb*). Urine-derived extracellular vesicles (EVs), because of its non-invasive sampling, lipid enrichment, and specific sorting character, have been recognized as a promising research target for biomarker discovery and pathogenesis elucidation in tuberculosis (TB). We aim to profile lipidome of *Mtb*-infected individuals, offer novel lipid signatures for the development of urine-based TB testing, and provide new insights into the lipid metabolism after *Mtb* infection.

**Methods:**

Urine-derived extracellular vesicles from 41 participants (including healthy, pulmonary tuberculosis, latent tuberculosis patients, and other lung disease groups) were isolated and individually detected using targeted lipidomics and proteomics technology platforms. Biomarkers were screened by multivariate and univariate statistical analysis and evaluated by SPSS software. Correlation analyses were performed on lipids and proteins using the R Hmisc package.

**Results:**

Overall, we identified 226 lipids belonging to 14 classes. Of these, 7 potential lipid biomarkers for TB and 6 for latent TB infection (LTBI) were identified, all of which were classified into diacylglycerol (DAG), monoacylglycerol (MAG), free fatty acid (FFA), and cholesteryl ester (CE). Among them, FFA (20:1) was the most promising biomarker target in diagnosing TB/LTBI from other compared groups and also have great diagnostic performance in distinguishing TB from LTBI with AUC of 0.952. In addition, enhanced lipolysis happened as early as individuals got latent *Mtb* infection, and ratio of raft lipids was gradually elevated along TB progression.

**Conclusion:**

This study demonstrated individualized lipid profile of urinary EVs in patients with *Mtb* infection, revealed novel potential lipid biomarkers for TB/LTBI diagnosis, and explored mechanisms by which EV lipid raft-dependent bio-processes might affect pathogenesis. It lays a solid foundation for the subsequent diagnosis and therapeutic intervention of TB.

## Introduction

Tuberculosis (TB) remains one of the most lethal infectious diseases in the world, posing a grave threat to public health. According to World Health Organization [WHO]’s (2023) global TB report, an estimated 10.6 million people were infected by *M. tuberculosis* (*Mtb*) and 1.3 million died from TB (World Health Organization [WHO], 2023). New TB cases in China for 2022 were approximately 748,000 with an estimated 30,000 deaths (World Health Organization [WHO], 2023). The burden for TB prevention and control was even more rigorous as a direct result of COVID-19 pandemic (Pai et al., 2022).

Currently, sputum smear and sputum culture are the gold standards for clinical TB diagnosis, which are time-consuming, less sensitive, and out of reach for many TB patients (Hepple et al., 2021). The limitations are even more apparent in children and the elderly, who cannot cooperate with sputum coughing for specimen collection. If methods for rapid testing by using body fluids can be developed, it would be a valuable addition to the diagnostic toolkit. Urine has been recognized as an ideal body fluid because it can be collected in large quantities without invasion, simple to handle, and low risk of infection when compared with other biofluids including serum, plasma, and saliva. In addition, urine-based TB testing is more affordable and device-free for allowing rapid TB diagnosis than blood-based testing (Hwang et al., 2022). In the past decades, high-throughput technology and multi-omic analysis have facilitated potential biomarker discovery in TB urine samples (Nogueira et al., 2022; Chen et al., 2023; Kim et al., 2023; Singh et al., 2023); we still face with great challenges for the disease-specific biomarker identification because of the huge number, broad range, and complexity of the components in human urine.

Extracellular vesicles (EVs), mainly exosomes, microvesicles (MVs), and apoptotic bodies, are nano-sized, double-membraned cup-like vesicles that were actively secreted from cells both under physiological and pathological conditions (Doyle and Wang, 2019). They can mediate signal transmission between cells by trafficking bio-active molecules including nucleic acids, proteins, and lipids (Singh et al., 2012; Lv et al., 2017; Lyu et al., 2019; Biadglegne et al., 2021). EVs are extensively present in various body fluids such as blood, urine, ascites, amniotic fluid, milk, cerebrospinal fluid, and bronchoalveolar lavage fluid (Singh et al., 2012; Biadglegne et al., 2021). Due to their lipid composition, EV lipids, apart from proteins and nucleic acids, have been recognized as a promising biomarker targets in a variety of diseases, including TB (Lam et al., 2021; Su et al., 2021; Yu et al., 2021). For instance, plasma exosomes from TB patients under different disease states (positive TB and TB lymphadenitis) showed distinct changes in the TAG and CE profiles compared with healthy controls, and their lipid spectrum was reorganized after TB treatment (Biadglegne et al., 2022). In addition, lipids are a key nutrient source for the growth and reproduction of *Mtb* (Maurya et al., 2019). Previous studies have indicated that *Mtb* can apply host fatty acids as their primary carbon source to enhance their survival *in vitro* and *in vivo* (Daniel et al., 2011; Maurya et al., 2019). However, research studies on lipidome of EVs from urine of TB have not been reported up to now. Therefore, revealing the lipid expression profile of urinary EVs from TB is of great value for biomarker discovery and pathogenesis elucidation.

This study examined the changes in lipid metabolism of the EVs derived from urine samples of healthy control (HC), latent TB infection (LTBI), tuberculosis (TB), and other non-TB lung diseases (Other) by using the targeted lipidomics technology platform (UPLC-TQMS). Furthermore, we analyzed and screened the characteristic lipid biomarkers for the TB/LTBI diagnosis. Finally, correlation analysis of lipids and lipid-associated proteins were carried out to obtain metabolic response networks in TB pathogenesis. Our study will offer novel lipid signatures with potential diagnostic value for the development of urine-based TB testing and provide new insights into the functions of disease-specific metabolism after *Mtb* infection.

## Materials and methods

### Participants and collection of samples

Urine samples from 41 participants including HC (healthy control, *n* = 10), LTBI (latent TB Infection, *n* = 7), TB (Tuberculosis, *n* = 10), and other (other lung diseases, *n* = 14, including 10 patients with lung cancer and 4 patients with pneumonia) were collected at initial diagnosis. All subjects were 18-year-old adults with HIV negative and signed the written informed consent form according to the Declaration of Helsinki, which was approved by the Ethics Committee of the Beijing Chest Hospital, Capital Medical University (number of ethical approvals: BJXK-2017-40-01). The HC individuals were recruited during annual health examination, and they have normal computed tomography (CT) chest films, negative tuberculin skin test (TST), and interferon-gamma release assay (IGRA) results. For LTBI individuals, they were diagnosed based on positive TST and IGRA results, but their other indicators were the same as healthy controls. All the TB patients were enrolled with positive *Mtb* culture and smear test results, and severe TB was identified with three or more lung lobe alterations observed in the CT scan and accompanied respiratory failure. Lung cancer and pneumonia patients were clinically newly diagnosed with negative IGRA results, and lung cancer patients had not received preoperative chemotherapy or radiotherapy. The clinical characteristics of the participants are shown in Supplementary Table S1.

### Urine-derived EV isolation and identification

EVs from urine were isolated by using exo-Easy Maxi Kit according to the manufacturer’s instructions (QIAGEN, Cat.76064). Transmission electron microscopy (TEM) was employed to visualize EVs using a JEM-1400 TEM (JEOL, Japan). Western blot was performed to detect EV surface marker CD9 and TSG101 with rabbit anti-human antibodies of anti-CD9 (SBI, United States) and anti-TSG101 (SBI, United States), and an endoplasmic reticulum marker protein, Calnexin, as negative control was detected with anti-Calnexin antibody (Abcam, United States). The size distribution of EVs was also measured through nanoparticle tracking analysis (NTA, Malvern, United Kingdom).

### Lipidomic analysis

Using high-performance liquid chromatography coupled with triple quadrupole mass spectrometry (UPLC-TQMS) (TACQUITY UPLCXevo TQ- S, Waters Corp., Milford, MA, USA), lipids from urine samples were detected and processed using MassLynx software (Waters, Milford, MA, USA) for peak extraction, integration, and quantification of each lipid, according to a set of standard samples of known concentration (Quantitative Curve, QC).

#### Profiling of lipidome in different groups

Heatmap was plotted based on z-scores of mean of lipid levels expressed by R.pheatmap (version 1.0.12), k-means clustering was performed to aggregate lipids that exhibited similar patterns of change, and lipids levels in each group were presented as line plots. Classes of the detected lipids were shown as follows: FFA, free fatty acid; LPA, lysophosphatidic acid; PA, phosphatidic acid; PE, phosphatidylethanolamine; PG, phosphatidylglycerol; CE, cholesterylester; Cer, ceramide; DAG, diacylglycerol; HexCer, Hexosylceramide; LPC, lysophosphatidylcholine; LPE, lysophosphatidylethanolamine; MA, monoacylglycerol; PC, phosphatidylcholine; SM, sphingomyelin; TAG, triacylglycerol; PS, phosphatidylserine; PI, phosphatidylinositol.

#### Differential expressed lipid analysis and biomarker selection

Multivariate statistical analysis of orthogonal partial least squares discriminant analysis (OPLS-DA) and univariate statistical analysis of *t*-test or Mann–Whitney–Wilcoxon (*U*-test) based on normality and chi-square of data were used to obtain differential expressed lipids (DELs) between two groups. Threshold value for potential biomarkers selection in this project is: VIP > 1 in multi-dimensional statistics and/or *p* < 0.05 and |log2FC| > = 0 in univariate statistics. SPSS version 13.0 software (SPSS Inc., Chicago, IL, USA) was used for receiver operating characteristic (ROC) curve analysis to evaluate the diagnostic performance of potential biomarkers. The following analysis was conducted using these DELs.

#### Analysis of lipolysis and ratio of raft lipids

Method used to illustrate TAG lipolysis, and the ratio of raft lipids was applied as Lam SM reported with minor revisions according to detectability and replaceability of lipid species based on our actual data: TAG lipolysis = TAGs/(DAGs+MAGs+FFAs); proportion of raft lipids = ((SM + CE)/PC) (Lam et al., 2021).

#### Lipid pathway enrichment analysis

Pathway enrichment analysis was carried out using the pathway-associated metabolite sets (SMPDB) with fold changes (FC) and *p*-value, and pathway analysis was performed using hsa set with pathway impact factor and *p*-value. Pathways with *p*-value<0.05 were plotted in this study.

#### Proteomic LC–MS/MS and correlation analysis of DELs and DEPs

protein samples (100 μg) from urinary EVs were digested and desalted and then dried by SpeedVac for mass spectrometry. LC–MS/MS was run on the EASY-nLC 1,000 (Thermo Scientific, USA) using an analytical column (C18, 1.9 μm, 75 μm*20 cm) at a flow rate of 200 nL/min. The *Homo sapiens* proteome sequence database containing 20,417 entries downloaded from UniProt was used for the database searching. Normalization was performed against the total peptide intensity of each sample. At least two mapping peptides were used as cutoff for protein identification. Statistical analysis of differential expressed proteins (DEPs) was assessed using t-test for pairwise comparisons. *p* < 0.05 and |log2FC| > = 0 were considered to be statistically significant. Among the DEPs, lipid-associated proteins were manually selected according to published data (Boerke et al., 2014; Sobocińska et al., 2018; Biadglegne et al., 2022; Franco Fontes et al., 2023). Correlation analyses (Pearson’s method) were performed on the selected lipid APs and DELs using the R Hmisc package (version 5.1–0).

## Results

### Characteristics of EVs from HC, LTBI, TB, and other groups

**Figure 1:**
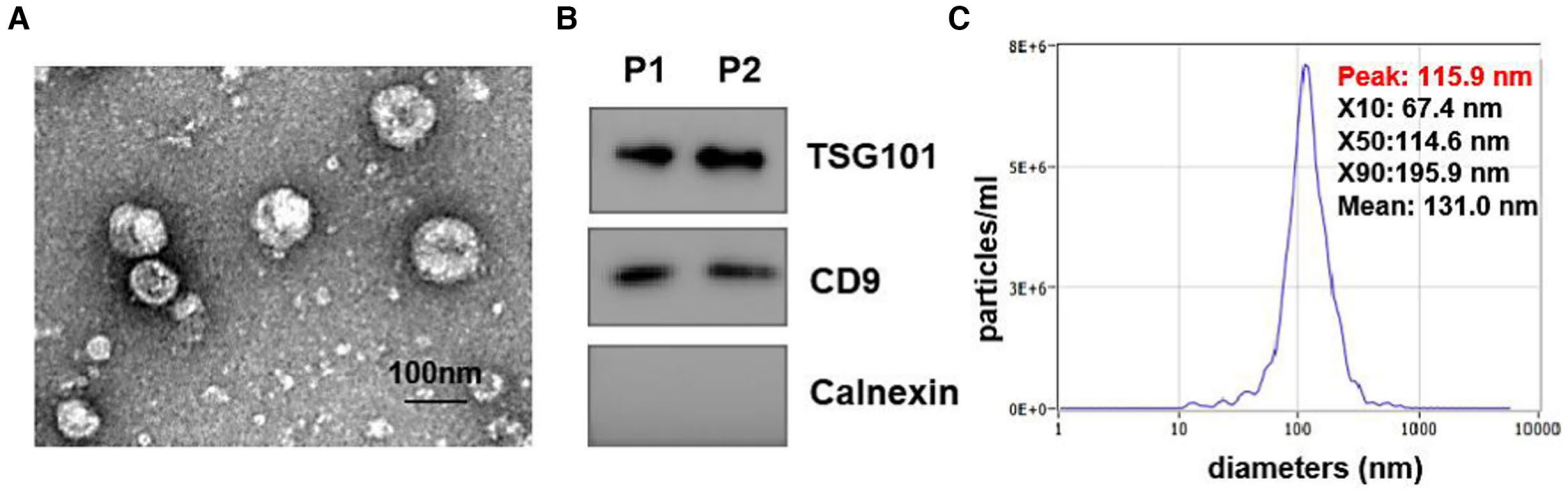
Identification of isolated urine-derived EVs. **(A)** Transmission electron microscopy (TEM) Image of EVs (magnification scale bar = 100 nm). **(B)** Western blot examined the EV surface marker of TSG101 and CD9, the expression of calreticulin (an endoplasmic reticulum marker protein) in vesicles. **(C)** Nanoparticle tracking analysis (NTA) showed the size distribution of isolated EVs, Y-axis present concentrations (particles/ml), the X-axis presented diameters (nm). The distribution peaks of EVs are at ~100 nm.

To explore the expression profiles of lipids in urine-derived EVs derived from HC, LTBI, TB, and other individuals, we isolated the urinary EVs using exoEasy maxi kit (Qiagen, 76064) and then visualized them by transmission electron microscopy (TEM) (Figure 1). A characteristic cup-shaped vesicle morphology was showed with the diameter approximately 100 nm (Figure 1A). Western blot analysis detected the expression of EV-specific markers of CD9 and TSG101 on them, with an endoplasmic reticulum marker protein Calnexin as negative control to exclude the contamination of cellular components (Figure 1B). The size distribution of the isolated EVs by nanoparticle tracking analysis (NTA) was in agreement with theoretical size of EVs ranging from 50 nm to 200 nm with the peaks at ~100 nm (Figure 1C).

### Lipid profiles and differential expression patterns in urine-derived EVs from HC, LTBI, TB, and other groups

**Figure 2:**
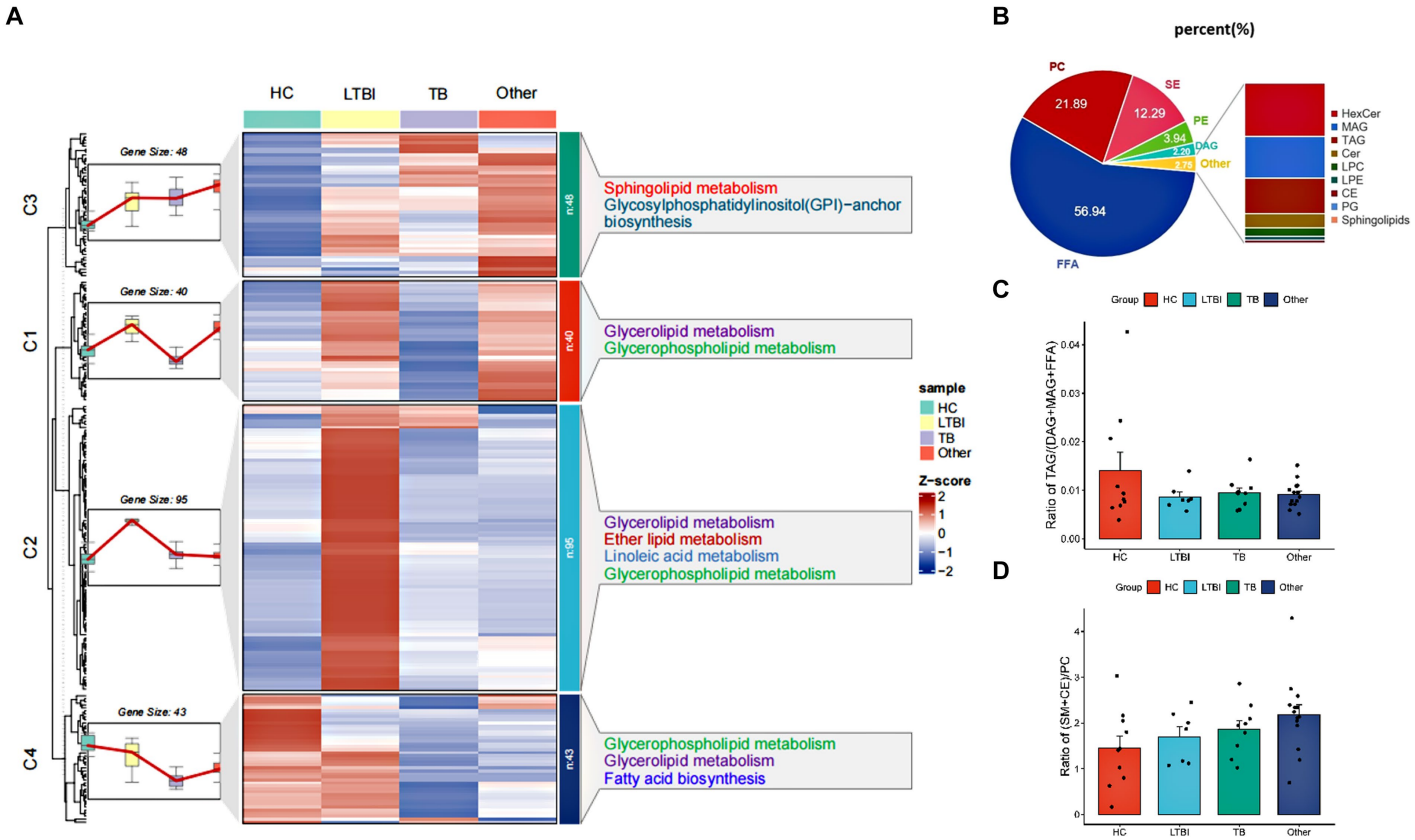
Profiling and different expression patterns of change in the lipidome of urine-derived EVs from LTBI and TB patients. **(A)** Heatmap was plotted based on z-scores of mean of lipid levels expressed, k-means clustering was performed to aggregate lipids that exhibited similar patterns of change in the LTBI, TB, an other. Lipids in four clusters were examined by KEGG Pathway Enrichment analysis. **(B)** Bar plot showed the distribution of different classes of lipids in each group. **(C)** Changes in the proportion of raft lipids ((SM + CE)/PC) across four groups. **(D)** Lipolysis was shown by the ratio of TAGs to their lipolytic substrates (DAGs) and free fatty acids (FFAs).

Using high-performance liquid chromatography coupled with triple quadrupole mass spectrometry (UPLC-TQMS), we profiled the lipidome of isolated urinay EVs from HC, LTBI, TB, and other groups (Figure 2A). Finally, up to 226 lipids belonging to 14 classes were detected among them with free fatty acids (FFA, 56.94%), phosphatidylcholine (PC, 21.89%), sphingomyelins (SM, 12.29%), phosphatidylethanolamine (PE, 3.94%), and diacylglycerol (DAG, 2.20%) as the top five classes (Figure 2B). Then, a global *k-*means clustering heatmap was constructed, and line plots were drawn on the basis of z scores of the mean of lipid levels, to illustrate the expression patterns of changes in the lipidome of EVs in different groups (Figure 2A). We showed that all the lipids were aggregated in four clusters with different KEGG pathway enrichment by functional and lipid gene enrichment analysis. Notably, there was the greatest proportion of lipids classified into clusters 1 and 2, in which LTBI had the most upregulated lipids compared with other control groups (Figure 2A). Moreover, as expected, glycerolipid metabolism and glycerophospholipid metabolism were the most enriched pathways across the clusters 1, 2, and 4, while substantial lipids were also enriched in KEGG pathway of sphingolipid metabolism in cluster 1 (Figure 2A). Accordingly, it has been reported that abnormalities in phospholipid, triglyceride, and sphingolipid metabolism were present in plasma exosomes derived from TB patients (Biadglegne et al., 2022). Interestingly, we noticed that pathway of glycosylphosphatidylinositol (GPI)-anchor biosynthesis was enriched in cluster 3, and it has been indicated that GPI-anchor proteins (GPI-APs) are key components of lipid raft, involving in key signal transduction such as T-cell activation and other immune responses (Simons and Toomre, 2000; Hou et al., 2016; Gagliardi et al., 2021).

To further address the lipolysis and raft lipid level in the tested groups, we examined the ratio of TAGs to their lipolytic substrates (DAGs) and free fatty acids (FFA), and it was reduced obviously in LTBI, TB, and other groups compared with HC, indicating enhanced lipolysis after *Mtb* infection and in individuals with lung diseases (Figure 2C). Moreover, we observed elevated proportion of raft lipids ((SM + CE)/PC) (Figure 2D) in the LTBI, TB, and other groups compared with HC, suggesting a role of lipid raft-mediated immune processes in TB pathogenesis and/or after lung injuries.

### Differentially expressed lipids in TB and LTBI indicate potential biomarkers for disease diagnosis

**Figure 3:**
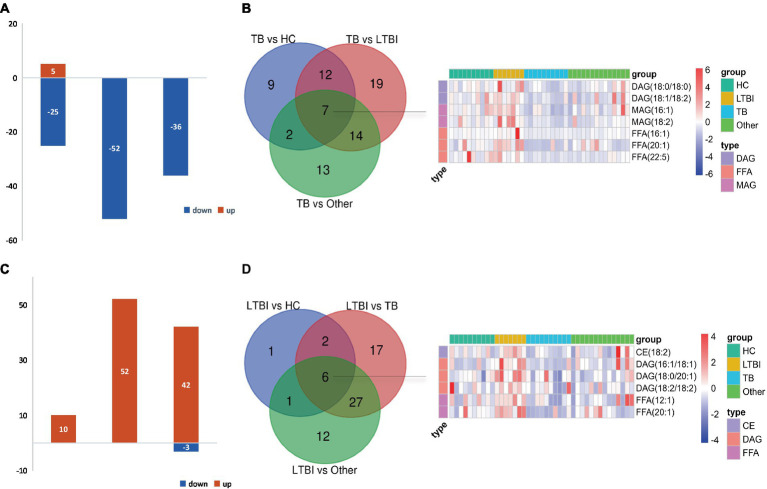
Differentially expressed lipids in LTBI/TB compared with control groups. **(A)** Bar plot showed significantly upregulated (red) or downregulated (green) lipids in TB. **(B)** Venn diagram presented overlapped lipids distinguishing TB from each control group and heatmap showed expression profile of overlapped seven lipids in each group. **(C)** Bar plot showed significantly upregulated (red) or downregulated (green) lipids in LTBI. **(D)** Venn diagram presented overlapped lipids distinguishing LTBI from each control group and heatmap showed expression profile of overlapped six lipids in each group.

**Figure 4:**
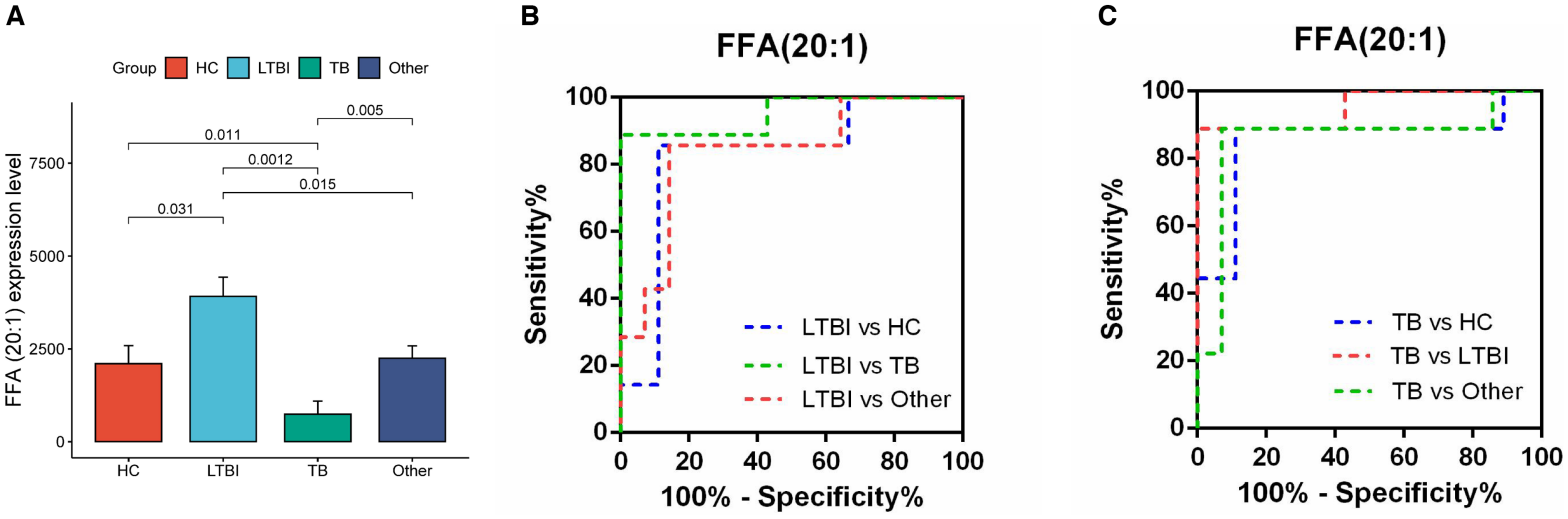
Diagnostic performance of lipid FFA (20:1) in distinguishing TB and LTBI from other control groups. **(A)** Expression level of FFA (20:1) in HC, LTBI, TB, and other, *p*-values were showed between LTBI/TB and each compared group. **(B,C)** Receiver operating characteristic (ROC) curve of the ability to detect FFA (20:1) in LTBI/TB and each compared group, respectively.

Next, we examined the differential expression profiles among the four groups by pairwise comparison based on OPLS-DA and univariate analysis (VIP > 1 and/or |log_2_(FC)| ≥ 0, *p* < 0.05) and identified 118 differentially expressed lipids (DELs) in TB and 107 DELs in LTBI compared with the other three control groups (Supplementary Figure S1; Figures 3A,C). Interestingly, 113/118 DELs were dramatically downregulated in TB, while 101/107 DELs were significantly upregulated in LTBI (Figures 3A,C). By constructing Venn plot, we identified seven overlapped DELs, including MAG (18:2), MAG (16:1), FFA (22:5), FFA (16:1), DAG (18:1/18:2), FFA (20:1), and DAG (18:0/18:0) in TB and six overlapped DELs including DAG (16:1/18:1), FFA (20:1), CE (18:2), DAG (18:0/20:1), DAG (18:2/18:2), and FFA (12:1) in LTBI, which could be served as potential biomarkers to differentiate TB or LTBI from other control groups (Figures 3B,D; Supplementary Tables S2, S3). Notably, FFA (20:1) was found in both TB and LTBI overlapped lipids, which has the remarkable expression in LTBI and the lowest levels in TB (Figure 4A). We then performed receiver operating characteristic (ROC) curve analysis to evaluate the ability to detect FFA (20:1) in LTBI/TB and each compared group. The AUC of FFA (20:1) in distinguishing LTBI from HC/other was 0.825 (specificity of 88.9% and sensitivity of 85.7%) and 0.837 (specificity of 85.7% and sensitivity of 85.7%), respectively (Figure 4B; Table 1). Meanwhile, the AUC of FFA (20:1) in diagnosing TB from HC/other was 0.852 (specificity of 88.9% and sensitivity of 88.9%) and 0.857 (specificity of 92.9% and sensitivity of 88.9%) (Figure 4C; Table 1). Importantly, the AUC of FFA (20:1) was 0.952, the highest in distinguishing TB versus LTBI, with specificity of 100% and sensitivity of 88.9%, respectively (Table 1).

**Table 1:** Statistics of FFA (20:1) distinguished TB/LTBI from compared groups.

Groups	AUC	95% CI	Specificity (%)	Sensitivity (%)	SE	*P-*value
LTBI vs. HC	0.825	0.597 to 1.054	88.9	85.7	0.116	0.031
LTBI vs. Other	0.837	0.645 to 1.029	85.7	85.7	0.098	0.015
LTBI vs. TB	0.952	0.848 to 1.057	100	88.9	0.053	0.001
TB vs. HC	0.852	0.648 to 1.055	88.9	88.9	0.104	0.011
TB vs. Other	0.857	0.664 to 1.050	92.9	88.9	0.099	0.005

### Characteristics of lipid expression changes along the different stages of *Mtb* (LTBI/TB) infection

**Figure 5:**
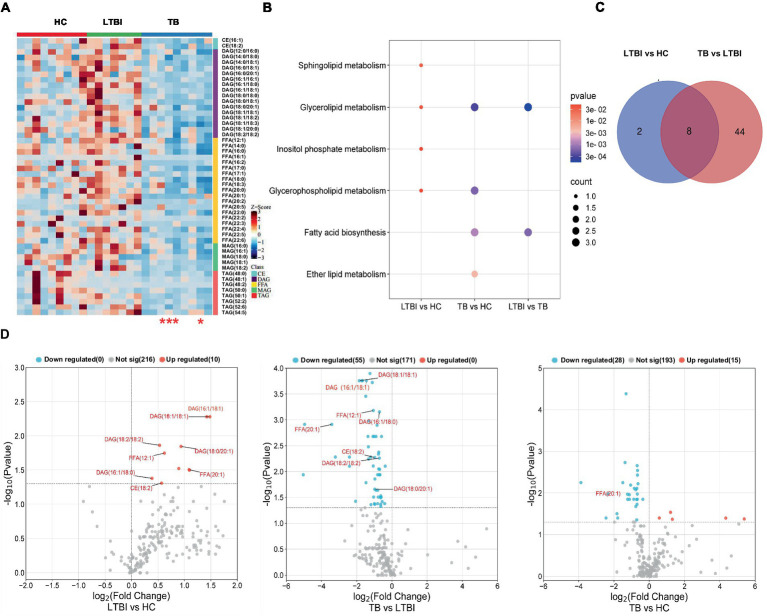
Distinct expression changes in lipids along the different stages of *Mtb* (LTBI and TB) infection. **(A)** Heatmap showing the differentially expressed lipids from HC, LTBI, and TB groups (VIP score > 1, p-value <0.05, |log2 (FC)| > 0). The color key indicates the expression level of the lipids. The four red stars mark severe TB patients. **(B)** KEGG pathway analysis and lipid enrichment were shown in compared groups (*p* ≤ 0.05). **(C)** Venn diagram showed the shared lipids altered over the courses of LTBI to TB. **(D)** Volcano plots showed significantly regulated lipids in compared groups. Red represented upregulation, blue represented downregulation. Shared lipids were labeled in red.

Our previous study has reported a gradually deteriorated progression with LTBI developing to the active TB (Lv et al., 2017; Lyu et al., 2019). Together with the probable dysfunction of lipid metabolism across TB progression, as shown in Figure 2, we then focused on the DEL profiles in HC, LTBI, and TB and their changes in LTBI versus HC and TB versus LTBI (Figure 5). DELs in both TB versus HC and LTBI versus HC were enriched in “glycerolipid metabolism” and “glycerophospholipid metabolism” (Figures 5A,B); besides, DELs in TB versus HC also played a role in “fatty acid biosynthesis” and “ether lipid metabolism,” while DELs in LTBI versus HC also gathered in specific pathways of “sphingolipid metabolism” and “inositol phosphate metabolism” (Figure 5B). Interestingly, there were 8 overlapped DELs between LTBI versus HC and TB versus LTBI, all of which were upregulated in LTBI but downregulated in stage of TB (Figures 5C,D). It showed that TB group has some differential regulation of lipids compared with HC rather than the changed lipids in progress of LTBI from HC (Figure 5D, the third graph).

### Correlation network of the differentially expressed lipids and lipid-related proteins in TB pathogenesis

**Figure 6:**
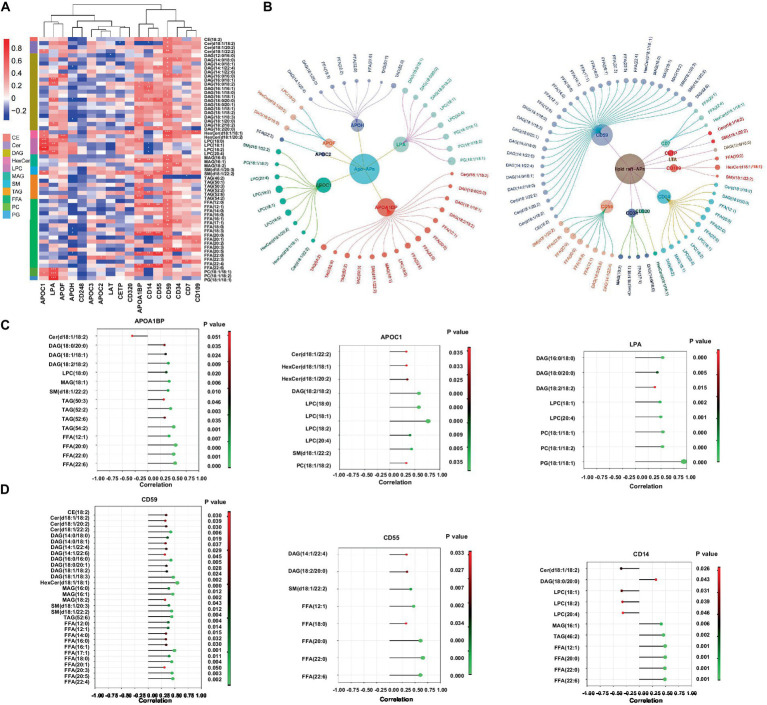
Correlation network of the differentially expressed lipids and lipid-related proteins in EVs from LTBI and TB. **(A,B)** Screened EVs apolipoprotein and associated proteins (Apo-APs) and lipid raft-associated proteins (lipid raft-APs) from LC–MS/MS proteome (unpublished data) were applied for Pearson correlation analysis with differentially expressed lipids. Inner dots presented proteins and outer dots presented lipids with larger sizes showing greater correlation coefficient (>0.3 or < −0.3). **(C)** Top 3 broadly correlated Apo-APs and **(D)** top 3 correlated lipid raft-APs and corresponding lipids were displayed with *p*-value (≤0.05) and correlation coefficient (>0.3 or < −0.3).

A combination of lipidomics and proteomics may identify the composition of EVs and reveal the metabolism specific to the parent cells of origin or discover unique biological functions that elicit a coordinated metabolic response at a system level (Xue et al., 2022). We then performed proteomics with the same samples for lipidomics and applied two omics data to perform correlation network analysis with all the DELs and DEPs identified in patients with *Mtb* infection. It has been reported that apolipoproteins were dysregulated in TB patients (Biadglegne et al., 2022). Meanwhile, there are some clues that lipid raft might play a role in TB progression; we thus screened lipid-related proteins from DEPs including apolipoprotein-asscociated proteins (Apo-APs) and lipid raft-asscociated proteins (lipid raft-APs) (Boerke et al., 2014; Sobocińska et al., 2018; Biadglegne et al., 2022; Franco Fontes et al., 2023) and constructed a correlation network which was displayed with *p*-value (≤0.05) and correlation coefficient (>0.3 or <−0.3) (Figures 6A,B). Among them, CD59, CD55, and CD14 were the top three highlighted lipid raft-APs with broader connections with DELs, and APOA1BP, APOC1, and LPA were the top three important Apo-APs (Figures 6C,D). Notably, nearly all of them were positively correlated with DELs in our experimental system (Figures 6C,D).

## Discussion

Lipids are significant components in the formation of foamy macrophages and tuberculous granuloma, both of which were favorable for the growth and reproduction of *Mtb* in host cells (Cronan, 2022; Florance and Ramasubbu, 2022). It has been reported that *Mtb* infection could lead to the accumulation of cholesteryl ester (CE) and triglyceride (TAG), promoted lipid droplet formation, and then used them as a long-term nutrition source (Daniel et al., 2011; Maurya et al., 2019). Previous studies also showed that mutations of genes in the specific pathway of lipid metabolism would recover the TB symptoms (Gioffré et al., 2005; Nazarova et al., 2017). Taken together, lipid homeostasis was of great value for TB research.

EVs are released from various human cells into the periphery, providing a potential source of tissue and disease-specific lipid biomarkers (Su et al., 2021; Yu et al., 2021). Our previous study has revealed potential miRNA and mRNA biomarkers for LTBI and TB by analyzing the transcriptome of their serum exosomes (Lv et al., 2017; Lyu et al., 2019). Importantly, we have identified exosomal genes from TB patients that enriched in GO terms of“lipid metabolism,” but the changes of the lipid metabolism in TB excreting EVs are largely unexplored (Lv et al., 2017). In the current study, we collected the promising testing sample of urine from TB patients and control groups and used large-scaled targeted lipidomic platform (UPLC-TQMS) to profile the distinct lipid expression spectrum in different stages of TB (LTBI and TB) and compared groups. Further analysis screened seven potential lipid biomarkers (downregulated) for TB and six lipid biomarkers (upregulated) for LTBI, all of which were classified into TAG lipolytic substrates (DAG, MAG), FFA and CE, in accordance with a recent study that reported decreased levels of certain types of TAG and FFA in the plasma of TB patients (Chen et al., 2021). Notably, we identified FFA (20:1) as the most promising biomarker target in diagnosing TB/LTBI from other compared groups with dramatic upregulation in LTBI but remarkable downregulation in TB. Specifically, it ranked the highest diagnostic performance in distinguishing TB versus LTBI with AUC of 0.952 (specificity of 100% and sensitivity of 88.9%). It has been demonstrated that FFA is accumulated in *Mtb*-infected macrophages, which mediates metabolic adaptation of host cells to anti-Mtb response and forces bacteria to become fat-fueled (Akaki et al., 2000; Taslim et al., 2017), explaining higher FFA (20:1) levels in LTBI at this critical turning point for the transition after *Mtb* infection but before active TB stage. Although there were several publications conducted lipid biomarker identification in TB prediction, diagnosis, or disease monitoring, all of them applied invasive plasma samples or plasma-isolated EVs, and there were also discrepancies for the classes and amounts of certain lipids among them and compared with ours (Chen et al., 2021; Biadglegne et al., 2022; Shivakoti et al., 2022; Anh et al., 2023). For example, by using UPLC–MS/MS, two lysophosphatidic acid (LPA) species of LPA (0:0, 16:0) and LPA (0:0, 18:0) in plasma were identified as potential biomarkers for early diagnosis and treatment efficacy of TB (Chen et al., 2021); however, LPA was not detected in our experiment system that might stem from different lipidomics platform and sample specimens. Meanwhile, another two metabolites of glycerophospholipid such as LPC and LPE were targeted by us but with no significant differences between TB and control groups.

Lipid metabolism was closely associated with host metabolic and immune alterations in TB pathogenesis (Shivakoti et al., 2022; Anh et al., 2023). Our results revealed distinct metabolism of glycerolipid, glycerophospholipid, and sphingolipid in LTBI and TB groups. In addition, the lipolysis presented by the ratio of TAGs/(DAGs+MAGs+FFAs) obviously increased from HC to LTBI stage and went slightly higher in TB stage, suggesting that enhanced lipolysis happened as early as individuals got latent *Mtb* infection so as to aid *Mtb* to survive dormancy (Daniel et al., 2011). In addition, the ratio of raft lipid was gradually elevated from HC to LTBI and then to TB, illustrating a potential role of lipid raft-associated process in TB progression. Lipid rafts refer to the formation of ordered, discrete microdomains enriched with cholesterol, sphingolipids, and GPI-anchored proteins within the membranes and has been discovered to be involved in the mechanisms of exosome biogenesis and secretion (de Gassart et al., 2003; Lingwood and Simons, 2010). So as to examine the biological processes related to lipid rafts, we further performed proteomics and conducted combinatorial analysis of lipidomics and proteomics (unpublished data) to build a correlation network between DELs and their related proteins such as Apo-APs and lipid raft-APs (GPI-anchored proteins) including CD59, CD55, and CD14 in lipid raft-APs and APOA1BP, APOC1, and LPA (APOA1) in Apo-APs, respectively. It has been reported that GPI-anchored proteins such as CD55 and CD59 were selectively sorted into exosomes (Rabesandratana et al., 1998). Their loss in red blood cells causes upregulated complement activation (Colden et al., 2022). CD59 was also required by pathogenic bacteria in cholesterol-dependent cytolysins by using virulence factors to punch holes in lipid bilayers (Parker and Feil, 2005; Boyd et al., 2016; Voisin et al., 2023). In addition, CD14 was also founded as immune activation markers on plasma exosomes from HIV patients (Chettimada et al., 2018). Recently, it was reported that trafficking of bacterial endotoxin lipopolysaccharide (LPS) by CD14 could modulate inflammatory responses of murine macrophages (Ciesielska et al., 2022). Thus, these GPI-APs, together with raft lipids, might be responsible for *Mtb* evading into cells and regulating EV-mediated immune signaling processes toward *Mtb*. As for lipids carrying apolipoproteins, APOA1, APOB, and APOC1 have been detected in plasma exoxomes from drug-resistant TB patients, and APOC1 was found to be deleted in patients after *Mtb* infection (Biadglegne et al., 2022; Wu et al., 2023). Consistently, we also identified LPA (APOA1)/binding protein (APOA1BP) and APOC1 in *Mtb*-infected patients, suggesting their possible involvement in the pathogenesis of TB by regulating lipid metabolism via EVs.

Taken together, our study has confirmed previous discoveries on aberrant lipid spectrum in circulating urine-derived EVs from individuals after *Mtb* infection, uncovered novel potential lipid biomarkers for TB/LTBI diagnosis with non-invasive sampling in a clinical setting, and revealed integral role of lipid metabolism and lipid raft-dependent processes in TB pathogenesis. However, some limitations in our work should be noted: firstly, we did not detect any *Mtb* virulent lipids such as reported sulfoglycolipid (SL) (Mishra et al., 2019), mannose-capped lipoarabinomannan (LAM) (Welin et al., 2008), trehalose dimycolate (TDM) (Spargo et al., 1991), glycopeptidolipids (GPL) (Sut et al., 1990), and phthiocerol dimycocerosate (PDIM) (Quigley et al., 2017) because they were not in the pool of our lipid targets, however, untargeted metabolomics applied by Biadglegne et al. did not report any *Mtb* lipids either, indicating their low levels beyond the detection capacity of current metabolomics platform. Second, the sample size of this study is relatively small, and further validation using larger, multi-center cohorts is needed to verify the diagnostic value of potential biomarkers for TB/LTBI and ravel the regulatory role of lipid-raft engaged biological processes in TB pathogenesis.

## Data availability statement

Metabolomics raw data have been deposited at EMB-MetaboLights (accession number MTBLS9666). Proteomics raw data have been deposited at ProteomeXchange (accession number PXD050345).

## Ethics statement

All the participants signed the written informed consent forms according to the Declaration of Helsinki, which was approved by the Ethics Committee of the Beijing Chest Hospital, Capital Medical University (number of ethical approvals: BJXK-2017-40-01).

## Author contributions

LL: Conceptualization, Data curation, Formal analysis, Funding acquisition, Writing – original draft, Writing – review & editing. HJ: Data curation, Resources, Writing – original draft. QL: Data curation, Funding acquisition, Writing – review & editing. WM: Formal analysis, Software, Writing – original draft. ZL: Data curation, Methodology, Writing – review & editing. LP: Methodology, Supervision, Writing – review & editing. XZ: Investigation, Methodology, Supervision, Writing – original draft, Writing – review & editing.
