# Supplementary material for: Individualized lipid profile in urine-derived extracellular vesicles from clinical patients with Mycobacterium tuberculosis infections
Source: Front Microbiol. 2024 May 30;15:1409552. doi: 10.3389/fmicb.2024.1409552 (PMC11169924; doi:10.3389/fmicb.2024.1409552)
Supplement: Supplementary file 1 [file Data_Sheet_1.DOCX]

**SUPPLEMENTARY MATERIALS**

**Figure S1 The differential lipids expression profile among the four groups.** Pairwise comparison based on OPLS-DA and univariate analysis (VIP>1 and/or |log2(FC)| ≥ 0, p < 0.05)


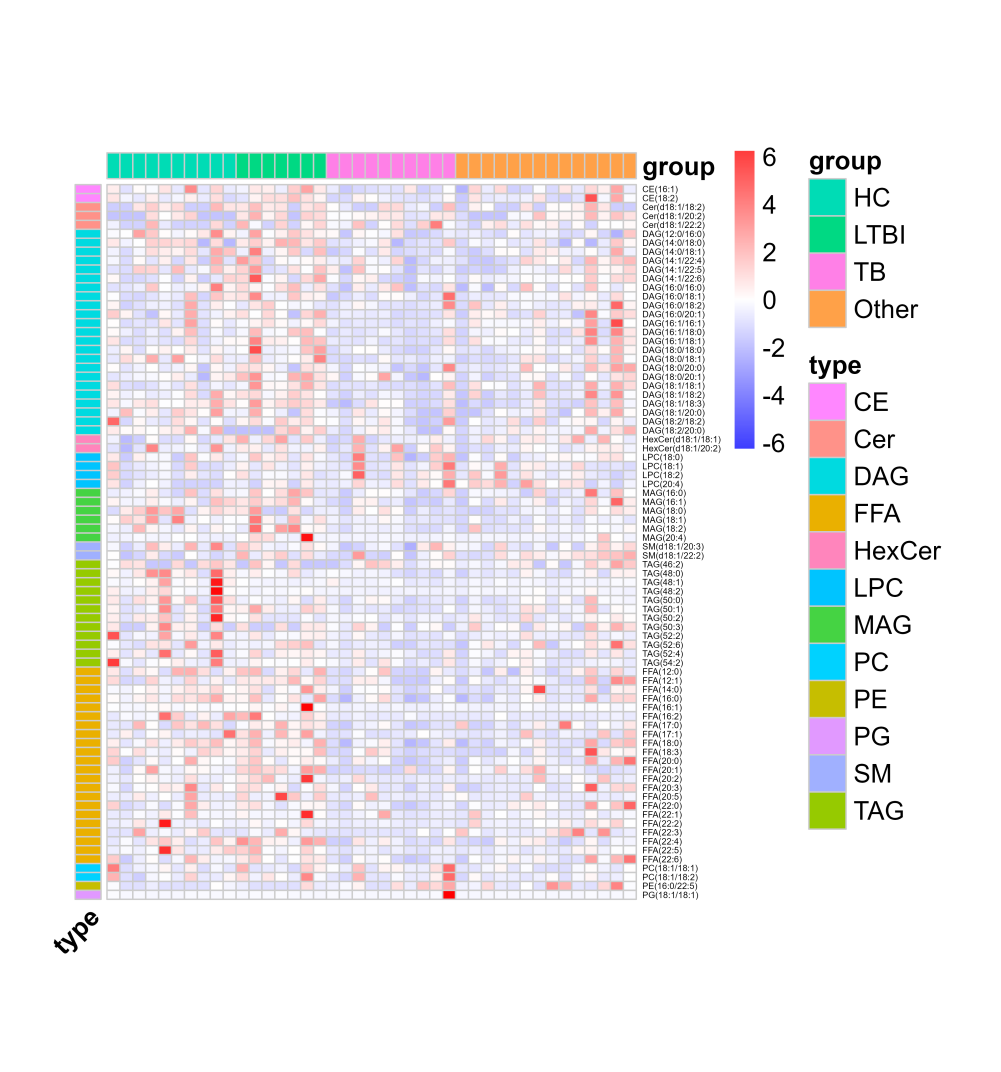


**Table S1 Basic demongraphic features of participants**

| **Characteristics** | **HC (n=10)** | **LTBI (n=7)** | **TB (n=10)** | **Others (n=14)** |
| --- | --- | --- | --- | --- |
| Median age±SD (range) | 48.7±2.8(43-53) | 52.7±4.9(43-57) | 53.3±11.9(35-69) | 54.1±12.8(32-72) |
| Gender(male/female) | 1/9 | 0/7 | 3/7 | 5/9 |
| Disease status | - | - | severe/non-severe (4/6) | lung cancer/pneumonia (10/4) |

**Table S2 Differentially Expressed Lipids in TB patients compared to control groups**

| **Lipids** | **Regulation** | **Adjusted *P*-value** | | | **Log2FC** | | |
| --- | --- | --- | --- | --- | --- | --- | --- |
|  |  | **LTBI vs HC** | **LTBI vs TB** | **LTBI vs Other** | **LTBI vs HC** | **LTBI vs TB** | **LTBI vs Other** |
| CE(18:2) | Up | 0.0491 | 0.0054 | 0.0099 | 0.3575 | 0.9724 | 0.9046 |
| DAG(16:1/18:1) | Up | 0.0052 | 0.0002 | 0.0031 | 0.8976 | 1.4522 | 1.0043 |
| DAG(18:0/20:1) | Up | 0.0143 | 0.0220 | 0.0031 | 0.5901 | 0.9813 | 1.6336 |
| DAG(18:2/18:2) | Up | 0.0137 | 0.0055 | 0.0354 | 0.3340 | 0.7155 | 0.4948 |
| FFA(12:1) | Up | 0.0178 | 0.0007 | 0.0002 | 0.3915 | 1.0547 | 0.9972 |
| FFA(20:1) | Up | 0.0311 | 0.0012 | 0.0340 | 0.6863 | 3.4166 | 0.7733 |

**Table S3 Differentially Expressed Lipids in LTBI compared to control groups**

| **Lipids** | **Regulation** | **Adjusted *P*-value** | | | **Log2FC** | | |
| --- | --- | --- | --- | --- | --- | --- | --- |
|  |  | **TB vs HC** | **TB vs LTBI** | **TB vs Other** | **TB vs HC** | **TB vs LTBI** | **TB vs Other** |
| DAG(18:0/18:0) | Down | 0.0143 | 0.0115 | 0.0342 | -0.7187 | -0.6770 | -1.3461 |
| DAG(18:1/18:2) | Down | 0.0106 | 0.0002 | 0.0196 | -0.6740 | -1.1141 | -1.5533 |
| MAG(16:1) | Down | 0.0211 | 0.0021 | 0.0112 | -0.7609 | -1.0772 | -1.3559 |
| MAG(18:2) | Down | 0.0142 | 0.0021 | 0.0005 | -0.6710 | -1.0132 | -0.8534 |
| FFA(16:1) | Down | 0.0000 | 0.0002 | 0.0213 | -1.3131 | -1.7102 | -1.1989 |
| FFA(20:1) | Down | 0.0106 | 0.0012 | 0.0101 | -2.3288 | -3.4166 | -2.4619 |
| FFA(22:5) | Down | 0.0056 | 0.0002 | 0.0148 | -1.4970 | -1.6708 | -1.2125 |
